# Knowledge Reuse of Multi-Agent Reinforcement Learning in Cooperative Tasks

**DOI:** 10.3390/e24040470

**Published:** 2022-03-28

**Authors:** Daming Shi, Junbo Tong, Yi Liu, Wenhui Fan

**Affiliations:** Department of Automation, Tsinghua University, Beijing 100084, China; tjb21@mails.tsinghua.edu.cn (J.T.); yiliu@mail.tsinghua.edu.cn (Y.L.); fanwenhui@tsinghua.edu.cn (W.F.)

**Keywords:** multi-agent, reinforcement learning, cooperative task, adding teammate, knowledge sharing, knowledge transferring

## Abstract

With the development and appliance of multi-agent systems, multi-agent cooperation is becoming an important problem in artificial intelligence. Multi-agent reinforcement learning (MARL) is one of the most effective methods for solving multi-agent cooperative tasks. However, the huge sample complexity of traditional reinforcement learning methods results in two kinds of training waste in MARL for cooperative tasks: all homogeneous agents are trained independently and repetitively, and multi-agent systems need training from scratch when adding a new teammate. To tackle these two problems, we propose the knowledge reuse methods of MARL. On the one hand, this paper proposes sharing experience and policy within agents to mitigate training waste. On the other hand, this paper proposes reusing the policies learned by original teams to avoid knowledge waste when adding a new agent. Experimentally, the Pursuit task demonstrates how sharing experience and policy can accelerate the training speed and enhance the performance simultaneously. Additionally, transferring the learned policies from the N-agent enables the (N+1)–agent team to immediately perform cooperative tasks successfully, and only a minor training resource can allow the multi-agents to reach optimal performance identical to that from scratch.

## 1. Introduction

The multi-agent system (MAS) is defined as a group of autonomous agents with the capability of perception and interaction. The multi-agent system has provided a novel modeling method for robot control [1], manufacturing [2], logistics [3] and transportation [4]. Due to the dynamics and complexity of multi-agent systems, many machine learning algorithms have been adopted to modify the performance of multi-agent systems, which is becoming an important factor of machine learning [5].

Multi-agent reinforcement learning (MARL) is a technique introducing reinforcement learning (RL) into the multi-agent system, which gives agents intelligent performance [6]. MARL achieves the cooperation (sometimes competition) of agents by modeling each agent as an RL agent and setting their reward. Multi-agent intelligence evolves relying on the exploration and exploitation of RL agents. However, it is the randomness of multi-agent exploration that makes it difficult for agents to finish cooperation tasks.

**Figure 1:**
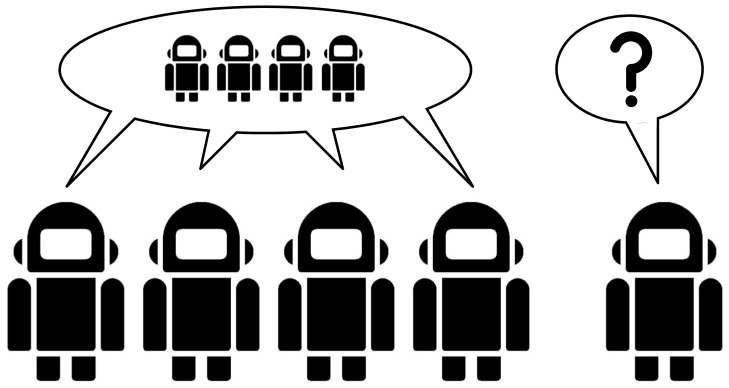
(1) Homogeneous agents are trained repetitively. Sharing knowledge among them is efficient and economical to train MARL. (2) MARL system is not robust to the dynamic variation in the number of agents. When adding a new agent, MARL system has to train all RL agents from scratch.

Current MARL algorithms keep multi-agents trained independently and repetitively, for the distributed intelligence of agents. However, the huge sample complexity of traditional RL methods is a well-known hindrance to applying both single and multi-agent RL in complex problems. The exponential growth of the state space with the number of agents usually requires prohibitive training resources. Especially in most cooperative tasks, homogeneous agents are all trained from scratch and might obtain different policies due to insufficient learning. Moreover, the MARL system is not robust to the dynamic variation in the number of agents, which results in the MARL system learning from scratch again. However, it is a common and necessary scenario to add a teammate for cooperative teamwork. Therefore, there are two problems of current MARL in cooperative tasks: (1) in most cooperative scenarios, many homogeneous agents are trained independently and (2) MARL requires learning from scratch when the system adds new agents, shown as Figure 1.

To cope with the above-mentioned problems, this paper proposes knowledge reuse methods for MARL in cooperative tasks of homogeneous agents. In the training procedure, the inter-agent knowledge-sharing algorithm is proposed by sharing experience and policy. The experiments demonstrate that sharing experience and policy can enhance the speed of training convergence and performance of cooperation. When the system adds a new agent, the inter-task knowledge reuse algorithm is proposed by reloading models from the original task. With the reuse of existing knowledge, the new multi-agent team could finish cooperative tasks immediately. Additionally, only a minor training resource could lead the new team with the added agent to reach comparable performance to learning from scratch.

This paper is organized as follows. Section 2 clarifies the related work of knowledge reuse in MARL. Section 3 proposes an inter-agent knowledge-sharing algorithm and an inter-task knowledge-transferring algorithm in cooperative tasks. Section 4 illustrates the experiments of our methods in the Pursuit domain. Finally, conclusions and future work are given in Section 5.

## 2. Related Work

Many researchers have shared experience and policy between agents to accelerate the training procedure of MARL. Tan proposed that sharing instantaneous information, episodic experience, and learned knowledge can speed up the training of agents [7]. Whitehead utilized External Critic and Learning By Watching to decrease the dimension of learning space [8]. L. Torrey and M.E. Taylor proposed a teacher–student algorithm to accelerate RL by consulting the teacher [9], and F. L. da Silva then proposed that students could only consult when they had uncertainty regarding the constraint bandwidth [10]. Souza L.O. proposed that experience of an unexplored region and experience of a high temporal-difference error should come prior to sharing [11]. These methods of sharing knowledge assume agents as having distinct identities and focus on how the experience and knowledge of predecessors accelerate the learning of successors. However, in this paper, we assume that the agents are all homogeneous and equivalent in all procedures of cooperative tasks. Therefore, agents should share their experience to explore more state–action space or share their policy to avoid obtaining different optimized policies in all procedures simultaneously.

Knowledge reuse within multi-tasking in the RL domain has also been extensively researched. Taylor researched the knowledge reuse of multi-tasking in a single agent with a full observation [12]. Glatt R. highlighted that transfer learning between similar tasks could accelerate RL training, while negative transfer should be avoided [13]. Omidshafiei researched the transfer learning of multi-tasking with a partial observation [14]. This research demonstrates how the similarity of tasks could accelerate the training procedure of new tasks. In this paper, the cooperation of the original multi-agent team and of a new team via the addition of agents constitutes two related tasks, whose similarity is the invariant cooperative target.

Recent researches utilize the ideology of sharing information or knowledge between multi-agents to promote cooperation. [15] proposes a sharing neural policy architecture for multi-agent on an autonomous vehicle coordination problem. Ref. [16] combines the cooperative sensing and multi-agent reinforcement learning to improve the sensing accuracy, by sharing spectrum detection. Ref. [17] implements a local wealth redistribution to promote cooperation of multi-agents. Ref. [18] researches multi-agents cooperating, where agents must learn to coordinate and share relevant information to solve the tasks. Ref. [19] proposes a partaker–sharer advising framework for cooperative MARL agents learning with budget constraints. Ref. [20] proposes that agents exchange information with their neighbors through a communication network to optimize the global cooperative return. Ref. [21] presents a cloud-native multi-agent platform allowing the transfer of the experience to the Internet of Things. Ref. [22] proposes a dual-arm to share their observations and actions to prevent the collision based on MARL. Ref. [23] enables satellites to share their decision policy to infer the decisions of others based on MARL. Ref. [24] develops a computationally efficient knowledge fusing mechanism to fuse the experience acquired by agents themselves and received from others. [25] proposes several methods for selecting experiences to accelerate the learning process.

It is a consensus that MARL lacks expansibility of the number of agents. There are two essential reasons for this, as follows: First, when the number of agents is altered, the dimension of the agents’ observation might be changed. This is infeasible for neural networks of deep RL or value tables of traditional RL. Second, the dynamic variant in the number of agents might lead to different optimal policies. Therefore, when the number of agents alters, it should be considered as a new task, given the same cooperative target. Currently, the Dynamic Agent-number Network (DyAN) designed by Wang W. enables the network to input data with different dimensions and tackle the problem of the changing number of agents [26]. In this paper, we fix the dimension of observation in cooperative tasks and research the reuse of the original policy and the learning of the current optimal policy, before and after adding new agents.

## 3. Methods

In this section, knowledge reuse methods of MARL in cooperative tasks are clarified. In this paper, we assume teammates can observe the position of others, but cannot communicate their actions. This assumption suits common scenarios, where the team observes the existence of all teammates while there is not a high demand for a communication bandwidth. Section 3.1 illustrates how multi-agents of MARL learn independently, share experience and share policies under the fixed number of agents. Additionally, Section 3.2 illustrates the knowledge transfer of new teammates and task transfer of new teams when a new agent is added.

### 3.1. Inter-Agent Knowledge Sharing in Cooperative Tasks

Section 3.1 clarifies the inter-agent knowledge-sharing algorithm by sharing experience and policies under a fixed number of agents. In this section, knowledge sharing occurs among agents in the same task.

#### 3.1.1. Independent Learning

Independent learning is the fundamental scenario in MARL, such as Team Q-learning [27] and Distributed Q-learning [28]. There are n agents in the environment. At any time, k, the agent, i, observes the state of the environment, $s_{i,k}\in S$, and chooses its action, $a_{i,k}\in A$. Additionally, agents will then receive a reward from environment, $r_{i,k}\in R=S\times A$. Here, i=1,2,3…n. Since we assume each agent can observe the position of others in cooperative tasks, we have $s_{i,k}=s_{k}$, i.e., every agent has full observation capacity. In full cooperative tasks of MARL, agents are usually given identical rewards at all times, $r_{i,k}=r_{k}$. However, the scarcity of such a setting will result in difficulty in convergence. In practice, part of the temporal reward is to help an agent learn. Additionally, the independent learning algorithm is shown in Algorithm 1.
**Algorithm 1:** Independent Learning1: 2: 3: 4: 5: 6: 7: 8: 9: 10: 11: 12: 13: 14: 15: 16: 17:initialize *n* RL agents with own replay buffer *RB_i_* **for** *episode* ← 1 **to** EPISODE    **for** *step* ← 1 **to** STEP         **for** *i* ← 1 **to** *n*
            Agent *i* chooses action *a_i_ = π_i_(s)*
        **end for**
        update state of environment *s’*= state(*s*, *a_1_*, *a_2_*...*a_n_*)        environment judges whether task is done d        **for**
*i*
 ← 1 **to** *n*
            Agent *i* gains reward *r_i_* = reward(*s*, *a_i_*, *s’*)            Agent *i* perceives experience (*s*, *a_i_*, *s’*, *r_i_*, *d*) into *RB_i_*        **end for**        **if** the task is done:            break this *episode*         **end if**    **end for** **end for**

**Figure 2:**
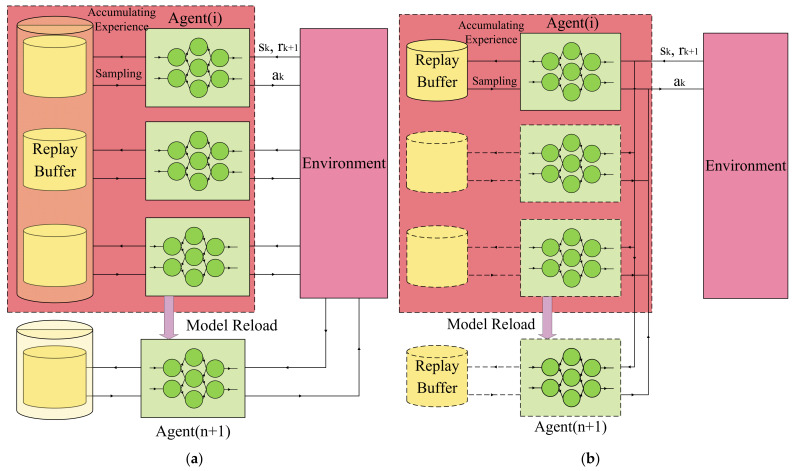
The framework of (**a**) experience sharing and (**b**) policy sharing of MARL. Agents share a common replay buffer of experience in (**a**) and share only one entity of policy network in (**b**). When a new agent is added into the system, the new agent reloads the model of the best (or shared) policy as initialization.

In independent learning algorithms of MARL in cooperative tasks, each agent observes the state of the environment (including the positions of others) and decides based on its own policy. Additionally, the environment will transit to the new state, s’, according to the actions of all agents $\left(a_{1},\;a_{2},\ldots ,a_{n}\right)$. Then, each RL agent accumulates the experience of this episode $e=\left(s,\;a_{i},s’,\;r_{i},d\right)$, which is deposited into their own replay buffer $RB_{i}$ and sampled randomly for the training and optimization of policy. This procedure is shown in Figure 2a. Here, the replay buffer of MARL enables the RL procedure to be offline learning rather than online learning [29].

#### 3.1.2. Experience Sharing

Reasonably, the agents of MARL in cooperative tasks are usually homogeneous. Their structure and properties are identical: the state set, S, of input and the action set, A, of output are identical, and their policies are based on the observation of the environment and other n−1 agents. Therefore, the experience of these homogeneous agents is also isomorphic and can be shared amongst one another. The concept of sharing experience has already been mentioned in [7,8,9], and we introduce this methodology into MARL in cooperative tasks, as shown in Algorithm 2.
**Algorithm 2:** Experience Sharing1: 2: 3: 4: 5: 6: 7: 8: 9: 10: 11: 12: 13: 14: 15: 16: 17: 18:initialize *n* RL agents with own replay buffer *RB_i_* **Initialize a common replay buffer RB** **for** *episode* ← 1 **to** EPISODE    **for** *step* ← 1 **to** STEP         **for** *i* ← 1 **to** *n*
            Agent *i* chooses action *a_i_ = π_i_(s)*
        **end for**
        update state of environment *s’*= state(*s*, *a_1_*, *a_2_*...*a_n_*)        environment judges whether task is done d        **for**
*i*
 ← 1 **to** *n*
            Agent *i* gains reward *r_i_* = reward(*s*, *a_i_*, *s’*)            Agent *i* perceives experience (*s*, *a_i_*, *s’*, *r_i_*, *d*) into **replay buffer RB**        **end for**        **if** the task is done:            break this *episode*             **end if**        **end for**    **end for**

In the experience sharing algorithm, although the experience, $e=\left(s,\;a_{i},s^{\prime },\;r_{i},d\right),$ is definitely generated and observed by the agent, i, this experience is also universal for other agents, as shown in Figure 2a. Therefore, all the agents contribute to the experience set and optimize their policies based on the common replay buffer. Experience sharing benefits the exploration of the state–action space and accelerates the convergence of policies.

#### 3.1.3. Policy Sharing

Although experience sharing speeds up experience accumulation and makes the exploration more intensive, the system is still optimizing n policies of agents, which confirms the MARL in the Curse of Dimension. To tackle such a problem, this section proposes the policy sharing of MARL in cooperative tasks. In the policy sharing of MARL, all agents share a common policy model in training and decision-making procedures, as shown in Algorithm 3.
**Algorithm 3:** Policy Sharing1: 2: 3: 4: 5: 6: 7: 8: 9: 10: 11: 12: 13: 14: 15: 16: 17: 18:**initialize a common agent *agent_share_* for *n* agents** **Initialize a common replay buffer RB** **for***episode* ← 1 **to** EPISODE    **for**
*step* ← 1 **to** STEP         **for**
*i* ← 1 **to**
*n*
            **Agent i chooses action *a_i_* = *π_share_*(*s*)**
        **end for**
        update state of environment *s’*= state(*s*, *a_1_*, *a_2_*...*a_n_*)        environment judges whether task is done *d*        **for**
*i*
 ← 1 **to**
*n*
            Agent *i* gains reward *r_i_* = reward(*s*, *a_i_*, *s’*)            ***Agent_share_* perceives experience (*s*, *a_i_*, *s’*, *r_i_*, *d*) into *RB***        **end for**        **if** the task is done:            break this *episode*
        **end if**    **end for** **end for**

In the policy sharing of MARL, the algorithm only maintains one reinforcement learning agent, $agent_{share}$. Due to the homogeneity of n agents, n agents training and optimizing their policies is equivalent to one common agent accumulating the experience of all agents and training its policy. Based on such policy sharing, one RL agent can make the decisions of all agents, as shown in Figure 2b. Apparently, policy sharing will dramatically decrease the time and space complexity and rid the multi-agent system of the Curse of Dimension.

### 3.2. Inter-Agent Knowledge Sharing in Cooperative Tasks

Section 3.1 illustrated inter-agent knowledge sharing (experience and policy sharing), with a fixed number of agents. In this section, we will propose how the system transfers knowledge when a new agent is added into the team: inter-task knowledge-transferring algorithm. In such a scenario, the original task is that n homogeneous agents perform a cooperative task. Additionally, the new task is that n+1 homogeneous agents perform such cooperative tasks.

#### 3.2.1. Policy Transferring of New Agent

The first problem to be tackled is where the policy or knowledge of the n+1 agent comes from when the system transfers from n to n+1 agents. If we assume that the policies of former n agents remain unchanged, then the former n agents are already able to perform a given task. Then, if the n+1 RL agent learns from scratch, the random exploration will disturb or interrupt the cooperation of former n agents. Therefore, the policy transferring of the n+1 agent is crucial for the new team.

In this paper, based on the greedy policy, we propose a system to explicitly achieve the most optimal policy of the former n agents. The realistic significance of this transferring is that the freshers are prone to replacing policies from sophisticated members (or the most optimal one). Therefore, in the three mentioned scenarios, the added agent will replace the policy model of the agent with the best performance in independent learning and experience sharing and replace the common policy model in policy sharing, as shown in the lower part of Figure 2.

#### 3.2.2. Task Transferring of New Team

When the former n agents hold original policies and the n+1 agent replaces one learned policy, the task of this new team has already been transferred, relative to these policies. In the original task, each agent will observe the state of the environment, n−1; its teammates; and itself, while in the new task, the observation includes the environment, n; its teammates; and itself, which transfers the input set of agents from S to S’. Moreover, the value functions of each agent, $Q\left(s,a\right)$, transfer to $Q’\left(s’,a\right)$ and the optimum of policy changes. Therefore, the optimal polices are not when the former n agents and the n+1  agent replace the original polices. Then, the multi-agent system requires an adaptation of the gap between original and new tasks, based on learned policies, as shown in Algorithm 4.
**Algorithm 4:** Inter-Task Knowledge Reuse1: 2: 3: 4: 5: 6: 7: 8: 9: 10: 11: 12: 13: 14: 15: 16: 17: 18***n* RL agents succeed the policies of original task** **the *n* +1 RL agent succeeds the best policy of original task** **for***episode* ← 1 to TRANSFER EPISODE    **for**
*step* ← 1 **to** STEP         **for**
*i* ← 1 **to**
*n*+1             Agent *i* chooses action *a_i_ = π_i_(s)*
        **end for**
        update state of environment *s’*= state(*s*, *a_1_*, *a_2_*...*a_n+1_*)        environment judges whether task is done *d*        **for**
*i*
 ← 1 **to**
*n*+1             Agent *i* gains reward *r_i_* = reward(*s*, *a_i_*, *s’*)            Agent *i* perceives experience (*s*, *a_i_*, *s’*, *r_i_*, *d*)        **end for**        **if** the task is done:            break this *episode*
        **end if**    **end for** **end for**

In the inter-task knowledge reuse algorithm, the original and new tasks are related but different. First, if each agent of n+1 agents ignore one of their teammates, they can still choose an optimal decision. This proves the original knowledge is beneficial to new tasks. Second, to gain the optimal policy of the new task, the transferring procedure is necessary, which takes up certain training resources.

Additionally, although the policies of original tasks are reused by the knowledge reuse algorithm, the experience set of the original task is deemed to be ignored. This is because the experience of the original task is the sampling of the original value function, where the state s∈S contains n agents and the environment. However, to optimize the new task, the sampling of the new value function is required, where the state of experience should be s’∈S’. Hence, the experience cannot be reused in the knowledge reuse of MARL in different tasks.

## 4. Results

In this section, we take the Pursuit task as an example to test the knowledge reuse algorithms of MARL. The Pursuit task was first introduced by Benda et al. [30], and the performances of different polices are clarified in detail. The Pursuit task is a classic example of cooperative tasks in the multi-agent domain and is widely employed by multi-agent researchers [31]. This section illustrates the scope of Pursuit and the performances of independent learning and experience sharing, and three policy-sharing scenarios, in both the basic Pursuit task and the agent addition task, are demonstrated, respectively.

**Figure 3:**
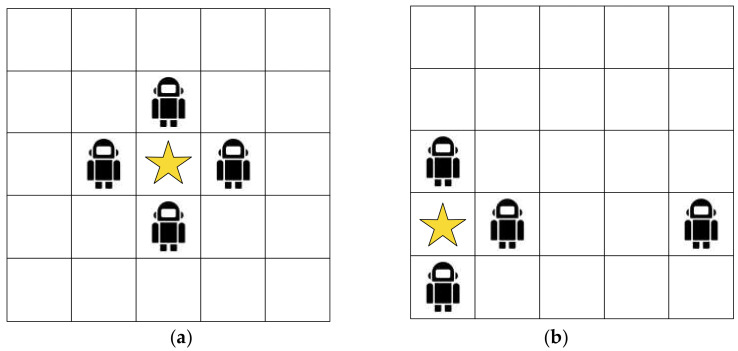
The Pursuit task: the star stands for the prey and the robots stand for predators. Subgraph (**a**) shows four predators block a prey successfully. Additionally, the state of subgraph (**b**) is equivalent to the left, since the grid world is torus. The star stands for the prey.

The traditional Pursuit task lives in a torus grid world, where moving off one side of the world brings the agent back onto the opposite side. There are four or five predators and one prey in the environment, and their actions consists of up, down, left, right and no-op movements. The predators win by capturing the prey, i.e., at least four predators block the prey at its four directions. The details of the Pursuit task are presented differently in the literature, and we simplify the task as follows: (1) Since the predators work near to the prey and the torus world extends infinitely, we assume the grid world has a 5 × 5 space, as shown in Figure 3. (2) The Markov decision process (MDP) is based on the grid world game, in which the prey moving in one direction is equivalent to all predators moving in the opposite direction. Moreover, if the prey is not in the centroid of the grid world, we can always translate all agents (prey and predators) to place the prey in the centroid explicitly, as shown in Figure 3. Hence, we can fix the prey in the centroid of the grid world without movements. While the predators initially show up randomly in the grid world to pursue the prey.

The hardware of the experiments is (Linux version 5.4.0-58-generic) Ubuntu 18.04, with CPU: Intel(R) Xeon(R) CPU E5-2620 v4 @ 2.10 GHz and GPU: NVIDIA GeForce RTX 2080. The reinforcement learning agents take deep Q-learning (DQN), one of the most classical deep RL algorithms [13]. The RL parameters include the training episode EPISODE = 20,000 and most experiment steps of each episode STEP = 50. The input of the RL agent is the 5 × 5 grid world, which keeps the input dimension constant when adding a new agent. Based on the above hardware and setting, we tested the independent learning, experience and policy sharing of MARL in both the basic Pursuit task and the agent addition task of Pursuit. The source codes of the following experiments can be downloaded on https://github.com/Daming-Shi/MARL_Pursuit (accessed on 7 March 2022).

### 4.1. Independent Learning

#### 4.1.1. Basic Task

**Figure 4:**
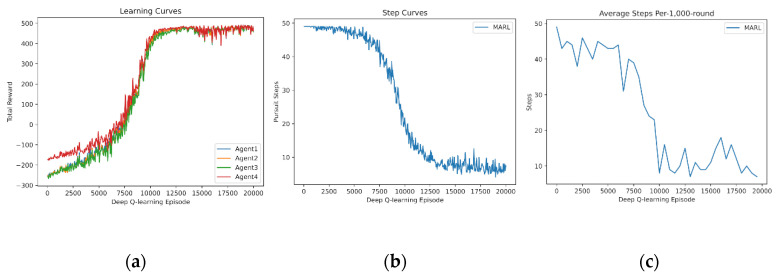
Independent learning of 4 agents from scratch: (**a**) reward curves, (**b**) Pursuit step curve, (**c**) Pursuit test of 1000 rounds.

In this subsection, we demonstrate the independent learning of MARL. First, four agents learn from scratch for Pursuit. The learning curve of the reward sum is drawn in Figure 4a. The learning curve of Pursuit step is drawn in Figure 4b. Additionally, the test curve of a fixed 1000-round Pursuit is drawn in Figure 4c, without exploration (i.e., no random actions). The learning curves demonstrate that four agents fail to capture the prey in the early procedure (0–5000 episodes). They quickly manage to capture it successfully and optimize the policy to reduce the step number rapidly in the medium procedure (5000–12,000 episodes). Finally, policies of Pursuit stabilize.

**Figure 5:**
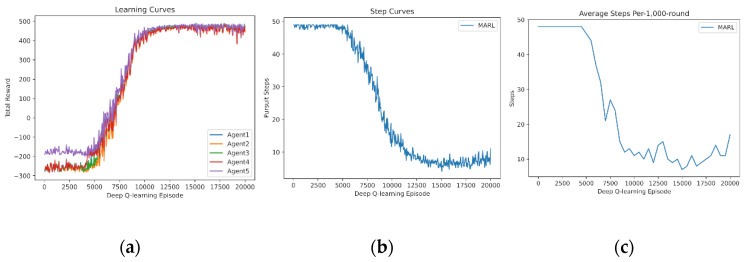
Independent learning of 5 agents from scratch: (**a**) reward curves, (**b**) Pursuit step curve, (**c**) Pursuit test of 1000 rounds.

Similarly, five agents learn from scratch and the results are drawn in Figure 5. It is found that five agents totally fail to cooperate with one another initially, because five agents have more disturbances than four agents do, which leads to greater failure. Additionally, as sufficient experience accumulates, five agents optimize the cooperative policies sharply and, therefore, the curves in the medium are steeper than those of four agents.

#### 4.1.2. Adding a New Agent

**Figure 6:**
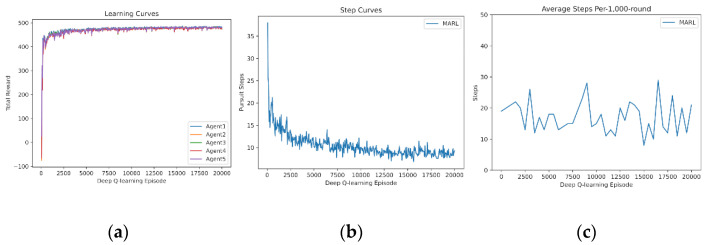
Independent learning of 5 agents with knowledge reuse: (**a**) reward curves, (**b**) Pursuit step curve, (**c**) Pursuit test of 1000 rounds.

As discussed above, learning from scratch when increasing the number of agents will waste the gained knowledge and training resources, and this paper proposes knowledge reuse to avoid learning from scratch. According to the algorithm in Section 3.2, the former four agents maintain their policies, and the added agent reloads the best policy of all of them. The experimental results show that five agents can capture the prey at the very beginning, with 18 steps. After the adaptation of 2500 episodes, the new team converges to a relatively good policy, effectively avoiding waste learning from scratch, as shown in Figure 6.

### 4.2. Experience Sharing

#### 4.2.1. Basic Task

**Figure 7:**
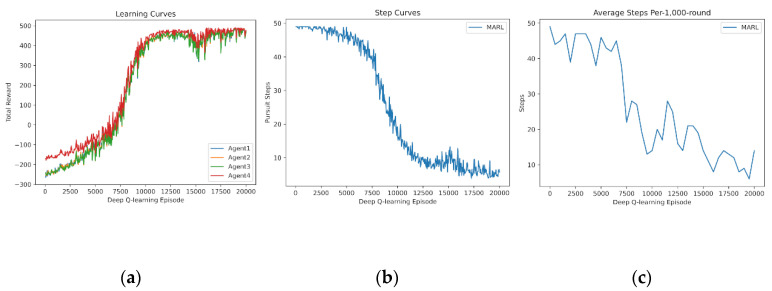
Shared experience learning of 4 agents from scratch: (**a**) reward curves, (**b**) Pursuit step curve, and (**c**) Pursuit test of 1000 rounds.

**Figure 8:**
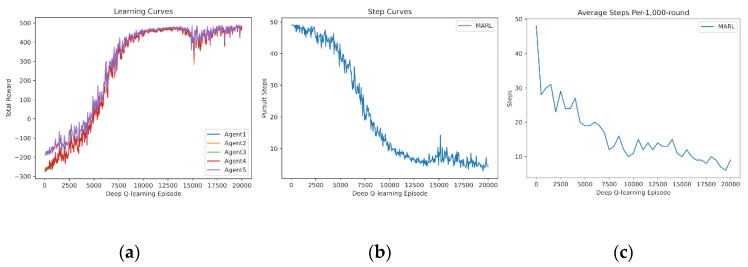
Shared experience learning of 5 agents from scratch. (**a**) Reward curves, (**b**) Pursuit step curve, (**c**) Pursuit test of 1000 rounds.

In this subsection, we demonstrate the experience sharing of MARL. The learning curves of four and five agents learning from scratch are drawn in Figure 7 and Figure 8. It is demonstrated that experience sharing can speed up the training procedure of MARL in cooperative tasks and decrease the rise time. Additionally, the curves obtain stable cooperative policies during 10,000–12,000 episodes. Additionally, the dip and vibrations at 15,000 episodes are caused by the overfitting of every random map. To suit for certain tasks or states, policy networks begin to overfit the training experience, which leads to declines in universality and scalability.

#### 4.2.2. Adding New Agent

**Figure 9:**
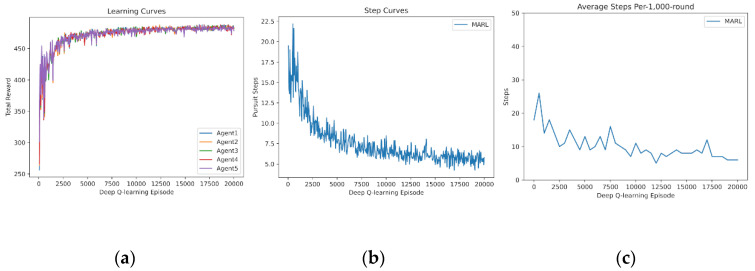
Shared experience learning of 5 agents with knowledge reuse: (**a**) Reward curves, (**b**) Pursuit step curve, (**c**) Pursuit test of 1000 rounds.

In the experience sharing scenario, we tested whether transferring gained policies can avoid waste learning from scratch. Nevertheless, the added new agent reloads the best policy of teammates, and the learning curves are shown in Figure 9. Policy transferring enables the five agents to initially succeed in capturing the prey and to quickly reach relatively good policies.

### 4.3. Policy Sharing

#### 4.3.1. Basic Task

**Figure 10:**
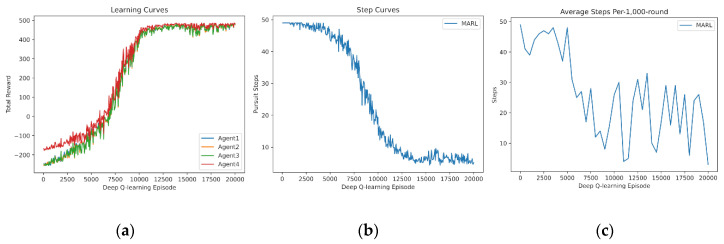
Shared policy learning of 4 agents from scratch: (**a**) Reward curves, (**b**) Pursuit step curve, (**c**) Pursuit test of 1000 rounds.

**Figure 11:**
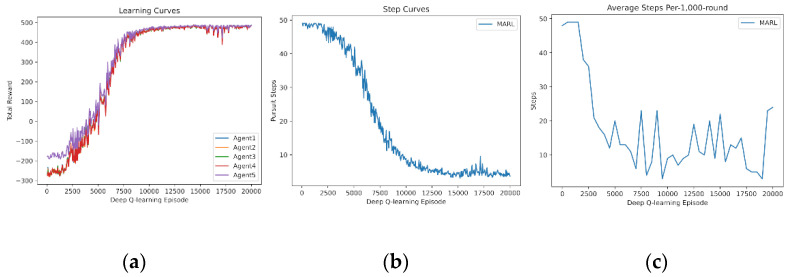
Shared policy learning of 5 agents from scratch: (**a**) Reward curves, (**b**) Pursuit step curve, and (**c**) Pursuit test of 1000 rounds.

In this subsection, we test the policy sharing of MARL. The learning curves of four and five agents learning from scratch are drawn in Figure 10 and Figure 11. Furthermore, policy sharing accelerates the training speed more than experience sharing and independent learning. Above all, the policy sharing scenario only maintains one policy network in all cooperative tasks, so policy sharing could effectively decrease the training time and storage space with a comparable performance to independent learning. Meanwhile, the network optimization is more likely to vibrate, as shown in Figure 10c and Figure 11c.

#### 4.3.2. Adding a New Agent

**Figure 12:**
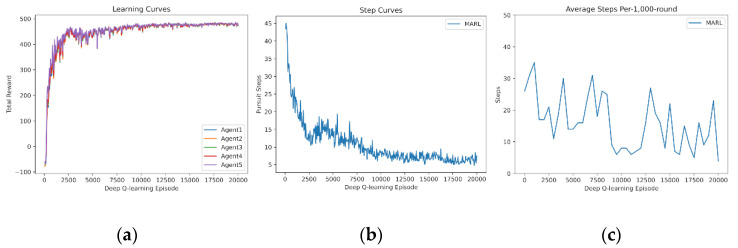
Shared policy learning of 5 agents with knowledge reuse: (**a**) Reward curves, (**b**) Pursuit step curve, and (**c**) Pursuit test of 1000 rounds.

Since all agents share a common policy network to make decisions in the policy sharing scenario, the newly added agent could explicitly share this policy. The learning curves are drawn in Figure 12. Again, policy transferring initially enables five agents to work and to quickly converge into a relatively good policy.

## 5. Discussion

**Figure 13:**
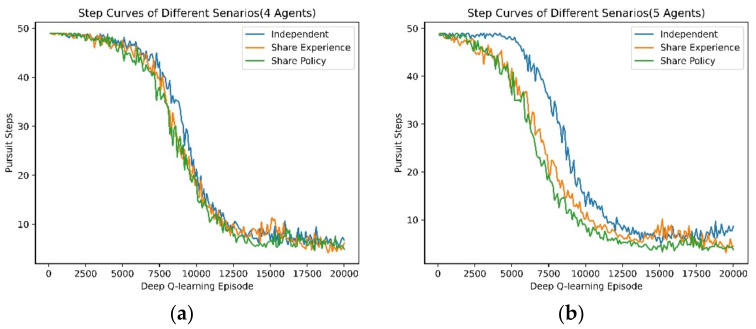
The average number of pursuit steps of independent learning, experience sharing and policy sharing: (**a**) 4 agents from scratch, (**b**) 5 agents from scratch.

This section analyzes the acceleration of experience and policy sharing in the training procedure of MARL. We drew the average number of pursuit steps of independent learning, experience and policy sharing, both in four agents and five agents, from scratch. It is demonstrated that knowledge sharing can increase the learning speed of MARL, in Figure 13.

On the other hand, we compared the optimal performances in different scenarios. Since the initial state of each episode in Pursuit is randomly generated, we calculated the optimal average and standard variance of pursuit steps in a fixed 1000 round pursuits, as shown in Table 1. In addition, we implemented a rule-based performance as a baseline, where each agent is assigned a target position initially and routes at the nearest direction.

In contrast, experience and policy sharing, proposed in this paper, could increase the performances and properties of multi-agent cooperation. The average and standard variance of pursuit steps of experience and policy sharing are smaller than that of independent learning. In particular, policy sharing could also decrease training and storage resources with a comparable performance.

In the scenario of adding a new agent, the knowledge reuse algorithm initially enables the multi-agent to reach a capable performance, and a minor training time could lead the policies to convergence. This strikes the balance of decreasing training costs and reaching acceptable performances. A minor training cost could allow the knowledge reuse algorithm to reuse the original policies, and therefore, avoid waste learning from scratch.

## 6. Conclusions

First, the inter-agent knowledge-sharing algorithm of MARL is proposed by sharing experience and policy in cooperative tasks. The inter-agent knowledge-sharing algorithm avoids the waste of homogeneous agents being trained independently and repetitively. Moreover, the training procedures of homogeneous RL agents are accelerated by sharing experience and policies. In particular, the policy sharing only maintains one policy network and decreases training and storage resources efficiently, which rids the multi-agent system of the Curse of Dimension. The Pursuit experiments demonstrate that experience and policy sharing could speed up the policy training and improve performance.

On the other hand, the inter-task knowledge-transferring algorithm of MARL is proposed to meet the demand for adding a new agent into the cooperative team. The newly added agent could replace the policy of an experienced teammate in the original task. Such policy transferring avoids the waste of the new team learning from scratch or disturbance from the added agent exploring randomly when adding a new agent. The Pursuit experiments demonstrate that the inter-task knowledge reuse algorithm could enable the multi-agents to cooperate effectively, and only a minor training resource could lead the new team with the added agent to reach comparable performance to learning from scratch. This provides a kind of method to reuse knowledge between the transition of adding a new agent in cooperative tasks.

With the development of autonomous robots in manufacturing, logistics and unmanned driving, the cooperation of the multi-agent is a crucial problem to raise productivity. According to the bandwidth of a realistic scenario, the MARL of cooperative tasks can choose to share experience and policy during the training procedure. Meanwhile, adding a new agent is also a possible scenario in a cooperative team. The inter-agent and inter-task knowledge reuse algorithms in this paper reuse the knowledge effectively and improve the performances and stability of cooperation. However, the knowledge reuse algorithms of MARL in this paper still assume the homogeneity of multi-agents and global observations. Additionally, how the heterogeneous multi-agents reuse experience and policies and how to overcome the challenges from partial-observations still require further study, which is also the future work of this paper.

## Figures and Tables

**Table 1:** The optimal average and stand variance of pursuit steps in a fixed 1000 round pursuits.

Scenario	Rule-Based		Independent Learning			Shared Experience			Shared Policy		
	4 Agent	5 Agent	4 from Scratch	5 from Scratch	5 with Knowledge	4 from Scratch	5 from Scratch	5 with Knowledge	4 from Scratch	5 from Scratch	5 with Knowledge
Avr	11.02	12.37	7.122	8.979	8.986	6.645	6.273	6.078	3.817	3.394	4.314
Std	17.392	18.678	10.649	13.851	9.273	10.933	9.931	11.251	3.624	6.426	5.429
